# Benefits of reversible contraception

**DOI:** 10.12688/f1000research.14370.1

**Published:** 2018-06-29

**Authors:** Helena Kopp Kallner

**Affiliations:** 1 Department of Clinical Sciences at Danderyd Hospital, Karolinska Institutet, Stockholm, Sweden; 2 Department of Obstetrics and Gynecology, Danderyd Hospital, Stockholm, Sweden

**Keywords:** Contraception, unintended pregnancy, abortion, LARC

## Abstract

Long-acting reversible contraception—intrauterine devices and contraceptive implants—offers the highest protection against unintended pregnancies. In addition, the use of reversible hormonal contraception has added health benefits for women in both the short and the long term. This review will give an overview of the benefits of reversible contraception as well as an evidence-based recommendation on how it should be used to benefit women the most.

## Introduction

In 2012, approximately 213 million pregnancies occurred worldwide ^1^. An unintended pregnancy is defined as a pregnancy that is unwanted or mistimed. In 2012, approximately 40% of all pregnancies were unintended, with 38% ending in live birth, 50% in abortion, and 13% in miscarriage ^1^. New data published in 2018 show that unintended pregnancies declined unevenly in the world, reflecting large differences in unmet need for contraception. In 2010–2014, 56% of unintended pregnancies ended in abortion, indicating that fewer women chose birth when confronted with an unintended pregnancy ^2^. Unintended and intended pregnancies alike may also result in the same proportion of miscarriage or ectopic pregnancy, which may result in subsequent subfertility or infertility.

It has been shown that long-acting reversible contraception (LARC)—intrauterine devices (IUDs) and contraceptive implants—offers the highest protection against unintended pregnancies. However, LARCs are rarely recommended for young women with high fertility. Short-acting reversible contraception (SARC) such as contraceptive pills, patches, rings, and injections are user dependent. Thus, the risk of getting pregnant because of inconsistent use is higher than for LARC.

In addition to protection against unintended pregnancy, the use of reversible hormonal contraception has added health benefits for women in both the short and the long term. The short-term added health benefits include a reduction of menstrual blood flow and protection against anemia, a reduction in dysmenorrhea and possible protection from symptoms of endometriosis, and a positive effect on mood in the premenstrual period. In the long term, all reversible contraception can be protective against some forms of cancer ^3^.

In recent years, new ways of using reversible contraception have extended benefits for women and improved acceptability. This review will give an overview of the benefits of reversible contraception as well as an evidence-based recommendation on how it should be used to benefit women the most.

## Protection against unintended pregnancy

In many parts of the world, unintended pregnancies arise from a lack of access to safe contraceptive methods. However, in parts of the world where efficient contraception is easily accessible, underutilization or user error is a common reason for contraceptive failure resulting in unintended pregnancy. Thus, reasons for unmet need of contraception vary in different settings. Most evidence on contraceptive use comes from women living in union (defined as traditional or legal marriage). Contraceptive use among young women not living in union in low-resource settings is less well known. Use of modern contraception was practiced globally by 57% of women living in union in 2015 ^4^. Therefore, presumably a large proportion of women globally have an unmet need for modern contraceptive methods.

LARC, such as subdermal implants and IUDs, results in significantly lower rates of unintended pregnancies owing to less user error compared to other methods, especially among young users who have lower user adherence ^5, 6^. LARCs also have higher satisfaction compared to SARC and higher continuation rates at 12 months ^7^. After an induced abortion, the use of LARC has been shown to reduce subsequent induced abortions due to unintended pregnancy ^8– 10^.

Although most women spend the majority of their fertile period trying to avoid pregnancy, the worldwide use of IUDs is estimated to be 14% ^4^, and the use of implants is much lower ^4^. In low-resource settings, the use of LARC is limited by access to health care professionals (HCPs) for placement and removal ^4^.

Among barriers to using IUDs in high-resource settings, fear of pain at insertion is commonly stated ^11, 12^. However, studies performed with an inserter 4.8 mm in diameter could not confirm that nulliparous women experience severe pain during insertion ^13, 14^. Recent studies have included insertion tube diameters of 3.8 and 4.8 mm. These studies confirm that less than 7% of women experience severe pain with the 4.8 mm insertion tube but that significantly fewer experience such pain with a 3.8 mm insertion tube ^14^. Thus, refraining from insertion of IUD in nulliparous women due to the patient’s fear of experiencing severe pain is not in line with current evidence, although there is still an ongoing debate.

### Ectopic pregnancy and the use of contraception

The focus on outcomes of unintended pregnancy tends to be on those that women can choose actively, i.e. abortion or birth. However, approximately 2% of all pregnancies are ectopic pregnancies ^15, 16^, which is the leading cause of maternal mortality in the first trimester and accounts for approximately 9% of maternal mortalities in the US ^16– 18^. LARC, such as IUDs, are user independent with a low risk of unintended pregnancy at 0.1–0.2% of women per user year ^6, 19, 20^. However, if pregnancy occurs, approximately 25–50% of these pregnancies are ectopic ^18^. Thus, LARC use reduces the risk of ectopic pregnancy compared to SARC use.

Risk factors for ectopic pregnancy include previous history of ectopic pregnancy, pelvic surgery, pelvic infections (pelvic inflammatory disease [PID]) such as sexually transmitted infections including chlamydia trachomatis, assisted reproductive technology, smoking, and use of intrauterine contraception ^17, 21^. These are generally not considered as contraindications for use of intrauterine contraception owing to the high contraceptive efficacy of the current devices and the failure rate of SARC due to inconsistent use.

### Methods for reducing risks of unintended pregnancy due to inconsistent use of pills, patches, and rings

The most common combination contraceptive pills and the available patch and ring all come with a hormone-free period of 7 days, during which women typically have a withdrawal bleed. Follicle growth starts on day 1 in the normal menstrual cycle. That follicle growth and ovarian suppression would therefore be affected by the length of the hormone-free period would seem logical, i.e. the longer time span without suppression, the larger the follicle grows and the closer the woman comes to ovulation. The benefit of a shorter hormone-free period has been previously suggested ^22^. Research shows that escape ovulation may occur with a 7-day hormone-free period ^23^. Although these escape ovulations may be due to inconsistent use of the method, they may also be due to a short menstrual cycle where ovulation may occur as early as 10 days into the menstrual cycle. In this study, the formulation with a 4-day hormone-free period produced a higher level of ovarian suppression. The higher level of ovarian suppression with a 4-day hormone-free period compared to a 7-day hormone-free period was confirmed in another trial where the size of the ovarian follicle was measured ^24^. Thus, recommending a maximum 4-day hormone-free period may be beneficial when counseling women who wish to avoid pregnancy. The effectiveness of pills in typical use has been shown to be higher with a hormone-free period of 4 days compared with 7 days ^25^.

## Short-term added health benefits with reversible contraception

### Reduction in menstrual bleeding

Although choice of method may influence bleeding patterns and menstrual pain, recent research also focuses on how different methods can be used for the best effects on health and well-being. Using contraceptive pills/rings or patches for an extended but defined period of time may be called extended or long-cycle use. Using the same methods for longer periods of time until bleeding occurs may be called flexible continuous use. Recently, a Cochrane review evaluated continuous or extended use versus routine cyclic use of combined hormonal contraceptives for contraception ^26^. The review was based on 12 randomized controlled trials and concluded that there was no difference in compliance between regular 28-day cycles or extended or continuous use nor was there any difference in discontinuation or discontinuation due to experienced bleeding problems. The extended or continuous regimens fared better in terms of headaches, genital irritation, tiredness, bloating, and dysmenorrhea.

A recent study compared three groups of women. One group adhered to a 24/4 cycle, a second group took a 4-day pill-free break when experiencing a 3-day bleeding episode, and a third group could choose when to have a 4-day pill-free break. It was concluded that women in the second group had the fewest bleeding days during 1 year ^27^. There is evidence that the combined hormonal ring may also be used in continuous or extended regimens similar to those of pills ^28^. It is therefore possible that advising women on how to use their combined hormonal contraception may contribute more to user satisfaction than which combined hormonal pill is prescribed. There are no prospective studies comparing continuous or extended cycle use with a 24/4 regimen in terms of contraceptive effectiveness.

Evidence suggests that a newer formulation of combined hormonal contraceptive pills with estradiol valerate reduces menstrual blood flow in women with heavy menstrual bleeding by as much as 90% when taken according to the manufacturer’s instruction ^29^ compared to a reduction of 43% with older formulations taken with a 7-day hormone-free period ^30^. Taking pills with estradiol and nomegestrol acetate reduces the number of cycles without any bleeding when compared to pills containing ethinyl estradiol and drospirenone if the pills are taken with a 24/4 or 21/7 regimen according to the manufacturer’s instructions ^31^. No comparative trials of bleeding patterns with extended or continuous regimens for pills containing estradiol/estradiol valerate or ethinyl estradiol have been published.

The most-effective method for reducing menstrual bleeding is the 52 mg levonorgestrel (LNG)-releasing intrauterine system (LNG-IUS) ^32^. In addition to providing women with a reduction in menstrual bleeding, the LNG-IUS provides women with a highly effective LARC. Until a few years ago, the only available LNG was the 52 mg LNG-IUS approved for 4 or 5 years of use depending on the brand. New evidence suggests that one of the 52 mg LNG-IUS may be used beyond 5 years ^33^. There are now two additional LNG-IUS with hormone capsules containing 13.5 mg and 19.5 mg LNG, respectively. The 13.5 mg LNG-IUS is approved for 3 years of use, whereas the 19.5 mg LNG-IUS is approved for 5 years of use. All LNG-IUS reduce menstrual bleeding. The 52 mg LNG-IUS is approved for the treatment of heavy menstrual bleeding and has been shown to reduce bleeding by up to 92% ^32, 34^. No such trials have been performed for the 13.5 mg or 19.5 mg LNG-IUS. In a randomized controlled trial comparing the three LNG-IUS, there were 23.6%, 18.9%, and 12.7% cycles without any bleeding in the 52 mg, 19.5 mg, and 13.5 mg LNG-IUS, respectively, at the end of the trial, which lasted for 3 years ^14^. This makes a dose-response relationship in bleeding pattern likely. No comparative trials have been published for the treatment of heavy menstrual bleeding with extended- or continuous-use regimens of combined hormonal contraception and the LNG-IUS.

### Premenstrual mood

It has been estimated that as many as 75% of women experience a negative effect on mood in the premenstrual phase and that approximately 3–5% of women have premenstrual dysphoric disorder with severe negative mood changes and experience social impairment because of this ^35^. The effect of hormonal contraception on premenstrual dysphoric syndrome/disorder has been investigated in reviews ^36^ and systematic reviews ^37^. These studies show that combined oral contraception improves premenstrual symptoms. A recent double blind randomized placebo-controlled trial of the effect on mood of a combined oral contraceptive containing 1.5 mg estradiol and nomegestrol acetate showed a significant improvement of mood in the premenstrual phase in the intervention group ^38^. However, this was not a trial on premenstrual dysphoric syndrome/disorder but with normal, healthy women. Healthy women with mild premenstrual symptoms may thus be advised that combined hormonal contraception may have a beneficial effect on mood during the premenstrual phase. In this trial, there was a non-significant worsening of mood in the early intermenstrual phase. However, the nocebo effect was pronounced, with as many as 17% of women in the placebo group and 24% of women in the intervention group indicating a worsening of mood during and after bleeding. Thus, a majority of women who indicate a worsening of mood may in fact experience a nocebo effect.

## Long-term added health benefits

### Effects on cancer

The effect of combined hormonal contraceptive pills on cancer has been studied in retrospective, prospective, and epidemiological studies. Results are partly consistent and partly contradictory. In 2017, the newest prospective data were published from the Royal College of General Practitioners’ oral contraception study, which recruited women in Great Britain in 1968 and 1969 ^39^. It concluded that ever use of combined oral contraceptives was associated with a reduced risk of colorectal cancer (incidence rate ratio 0.81; 99% confidence interval [CI] 0.66–0.99), endometrial cancer (incidence rate ratio 0.66; 99% CI 0.48–0.89), and ovarian cancer (incidence rate ratio 0.67; 99% CI 0.50–0.89). In addition, a reduction in lymphatic and hematopoietic cancer (incidence rate ratio 0.74; 99% CI 0.58–0.94) was seen. Several studies have shown an increase in breast cancer during current use of combined hormonal contraception, which was confirmed in a meta-analysis of these studies ^40^. However, this risk could not be confirmed in the Oxford Family Planning Association oral contraceptive study ^41^. However, those studies that showed an increased risk also showed that this increased risk does not persist 5–15 years after stopping combined hormonal contraception, whereas the reduction in risks of ovarian, endometrial, and colon cancer persisted ^39, 42, 43^. In addition, no new increased cancer risks appeared later in life in any of the studies. In the large prospective Nurses’ Health study with results published in 2014, it was concluded that groups with use or non-use of combined oral contraception did not differ in all-cause mortality ^42^. In all large prospective studies, women were married and cohabitating. Women in the Nurses’ Health study were 30–55 years old and nurses, whereas women in the Royal College of General Practitioners’ oral contraception study had a median age of 29 at recruitment. Women in the Oxford Family Planning study were aged 25–39. In addition, the majority of women used oral contraceptives with 50 mcg of ethinyl estradiol, which is a higher dose than commonly used today. However, results are reassuring for women starting combined hormonal contraception today.

A recent large epidemiological study from Denmark studied the effect of modern contraceptives on breast cancer ^43^. The study showed similar results to previous studies in increased breast cancer risk for women with combined hormonal contraception. However, the study showed that current or recent LNG-IUS use was also associated with a small but significant increase in receiving a diagnosis of breast cancer with a relative risk of 1.21 (95% CI 1.11–1.33). The 52 mg LNG-IUS has been shown to be effective in the prevention of endometrial cancer ^44^. There are currently no studies which have studied the long-term effect of LNG-IUS or low- or medium-dose progestin methods on ovarian or colon cancer risk, as these methods have not been on the market long enough.

## Conclusion

Women in high- as well as low-resource settings experience the social, economic, and health consequences of unintended pregnancies. High sterilization rates limit women’s reproductive choices and deny them access to the added benefits of reversible hormonal contraception. Apart from abortion and birth, which are options women may choose, unintended pregnancy may also result in ectopic pregnancy, which may threaten health and the future fertility of women. Women, and to some extent HCPs, are in many cases unaware and need increased information on the differences in effectiveness of different contraceptive methods and how methods should be used to improve effectiveness in typical use.

Fear of negative health effects of hormonal contraception remain an obstacle to the use of effective contraception for many women. Women fear a true but very small increased risk of breast cancer but remain ignorant of the many proven added health benefits of hormonal contraception, such as reduction in menstrual blood flow and menstrual pain and protection against certain cancers. There are also notions that the long-term health effects of hormonal contraception are somehow unknown and unexplored because of a lack of research. However, there are several large prospective, retrospective, and epidemiological studies of hormonal contraception which clearly show that these fears are exaggerated.

New large prospective studies on contraceptive use and especially the use of LNG-IUS and medium-dose progestin-only contraception and their effects on cancer and mood are lacking. Efforts should be made to carefully design these studies so that confounders can be controlled.

Reversible hormonal contraception has short-term as well as long-term benefits for women which deserve more attention from HCPs and women alike. If women are provided with more nuanced information regarding the risks and benefits of effective reversible contraception, they may make more informed decisions on the use of effective contraception and gain a higher quality of life.
